# Mercury Bioaccumulation in Mangrove Oysters (*Crassostrea rhizophorae*) (Guilding, 1828) and Associated Human Exposure from the Parnaíba River Delta, Equatorial Coast of Brazil

**DOI:** 10.3390/toxics13080678

**Published:** 2025-08-14

**Authors:** Thays Thayanne Luz-Santos, Victor Lacerda Moura, Moisés Fernandes Bezerra, Luiz Drude de Lacerda

**Affiliations:** Laboratório de Biogeoquímica Costeira, Instituto de Ciências do Mar-Universidade Federal do Ceará, Av. Abolição, 3207, Meireles, Fortaleza 60165-081, CE, Brazil; thays.santos1599@gmail.com (T.T.L.-S.); victuh00@gmail.com (V.L.M.); mmoisesfb@gmail.com (M.F.B.)

**Keywords:** mercury, biomonitoring, human exposure, oysters, mangrove

## Abstract

The present study quantifies mercury (Hg) concentrations in mangrove oysters (*Crassostrea rhizophorae*) and assesses their potential as biomonitors of Hg contamination in the Parnaíba River Delta (PRD), located on the equatorial coast of Brazil (ECB). The highest Hg concentrations occurred in the smallest individuals’ size class (20–40 mm) from the main channel of the Parnaíba River (52.1 to 195.4 ng g^−1^ w.w.), whereas the largest individuals’ size class (larger than 60 mm) exhibited the lowest Hg concentrations (35.2–114 ng g^−1^ w.w.). There was a significant correlation between Hg concentrations and shell sizes, either when considering all size classes or when considering only individuals of size classes smaller than 40 mm. Oysters larger than 40 mm did not present any significant correlation between Hg concentrations and size. In addition to size, higher concentrations were observed at the freshwater–seawater transition in the main channel sites. These maximum suspended particulate zones, with bottom sediment resuspension, can favor Hg adsorption to fine particles, increasing the bioavailability of Hg. A regional comparison of Hg concentrations in mangrove oysters from the ECB suggests they are efficient biomonitors at a regional level. In contrast, the environmental dynamics of the PRD, with high variability within sites, hampered its use at the local level. Mangrove oysters from the PRD are shown to be safe for human consumption, as far as Hg exposure is concerned, and the presented risk assessment shows no excessive exposure, even at high-frequency consumption rates.

## 1. Introduction

Mangroves make up a conspicuous component of highly complex transitional coastal zones in the tropics and subtropics, which comprise about 0.7% of all tropical forests. Mangroves provide important socioeconomic and ecological services, mainly associated with the conservation and sustainable uses of the wide variety of biota that permanently inhabit or visit mangroves, using them as a nursery and/or food source [1]. This ecosystem efficiently traps and retains most chalcophile metals in its soils, including toxic pollutants, such as mercury (Hg). However, due to soil metabolism-based sulfate reduction and the high productivity and exportation of dissolved organic carbon (DOC), mangrove ecosystems can also be a source of metals capable of strongly binding to DOC, including Hg [2]. The various mechanisms by which mangroves may act as sinks or sources of metals are detailed in Lacerda et al. [3,4]. Therefore, even relatively pristine mangroves may attain relatively high Hg concentrations in their inhabitant aquatic biota.

Mercury is a toxic element and one of the most concerning global contaminants due to its toxicity and persistence in the environment. Natural sources of Hg include volcanic eruptions, geothermal springs, geologic deposits, and the weathering of rocks [5], but anthropogenic sources, including land use changes, currently contribute to about 50% of the total Hg emission to the biosphere [6]. In Northeast Brazil, the main anthropogenic sources of Hg in estuaries are untreated urban effluents, inadequate disposal of solid wastes, intensive shrimp farming (as a ubiquitous component of aquafeeds based on fish meal), fuel and biomass burning, and, in some specific sites, small-scale artisanal gold mining and chlor-alkali plants [7,8,9]. In the Parnaíba river Delta (PRD), natural processes (atmospheric deposition and soil weathering) are responsible for most Hg emissions, although inadequate disposal of urban wastes, biomass burning, and intensive shrimp farming are human activities present in the basin and also contribute some anthropogenic Hg emissions [10,11]. Therefore, the PRD continues as a pristine delta that presents low human influence relative to Hg contamination, with Hg concentrations in water, sediments, and suspended matter relatively low compared to other estuaries on the NE coast of Brazil [12].

The availability of Hg in aquatic systems is of interest due to its bioaccumulation and biomagnification capacity through the aquatic food chain, increasing its concentrations from primary producers to consumers. Bivalves are widely distributed in estuaries worldwide and, as filter-feeding animals, may ingest both phyto- and zooplankton and thus occupy the second or third trophic level. The immobility of filter feeders and their high capacity to filter suspended particles allow them to accumulate both essential and nonessential trace elements in their tissues [13,14]. The mangrove oyster *Crassostrea rhizophorae* (Guilding, 1828) is a typical filter-feeding bivalve distributed along coastal zones in the Caribbean and Brazil and is particularly abundant in the PRD, with high commercial importance as fisheries [15].

Brazilian production of *C. rhizophorae* was about 173.89 tons in 2022, corresponding to about 1.8% of total mollusk production in this country [16]. According to this official report, mangrove oysters harvested in the Northeast region account for more than 76% of the total production of this species. For the PRD, mangrove oyster production is about 750 kg day^−1^ of wild-caught artisanal harvesting. Monthly production varies with season from 7.2 to 14.4 tons; about 350 kg month^−1^ of oyster meat [17]. In 2014, unfortunately the last statistics available, an annual total of about 91 tons of oysters (about 5000 kg of meat) was commercialized in the local fishers’ harbors of the PRD [15]. Despite the lack of official estimates, the consumption of mangrove oysters is a part of local tradition, especially in the coastal states of Brazil [18].

From an environmental monitoring perspective, several studies highlight the potential advantages of using oysters for the monitoring of environmental pollution and toxicological risks at different geographical scales [19,20]. The known bioaccumulation of Hg in oysters allows for extensive surveys of Hg concentrations and their speciation in estuaries, particularly in mangrove wetlands [21]. The use of oysters as biomonitors and as sentinel species of metal concentrations in estuarine environments has also been largely used in Brazil in past years [19,22,23]. However, studies involving *C. rhizophorae* on the equatorial coast of Brazil (ECB) are more restricted to estuaries in the eastern portion, extending from Ceará to Pernambuco states [7,24,25,26]. Despite this, there are no reports on Hg bioaccumulation in oysters from the PRD, notwithstanding its large production, economic importance, and the overall ecological significance of the PRD.

The PRD presents a rich biodiversity due to its pristine environmental conditions providing fisheries resources, such as oysters, which provide an important source of protein and income for the local human communities [27]. Because of the high ecological significance of the PRD and the importance of its fisheries, Hg contamination can pose a threat to food safety. Thus, the present study, understood as a pilot, since no previous record of Hg concentrations in the PRD oysters exists, aims to quantify, for the first time, Hg concentrations in mangrove oysters (*C. rhizophorae*) and human exposure to Hg through oyster consumption. In addition, the study evaluates the potential of these organisms as biomonitors of Hg on the local and regional scales by comparing oysters collected in different stations within the PRD and data available from other locations along the ECB.

### Study Area

**Figure 1:**
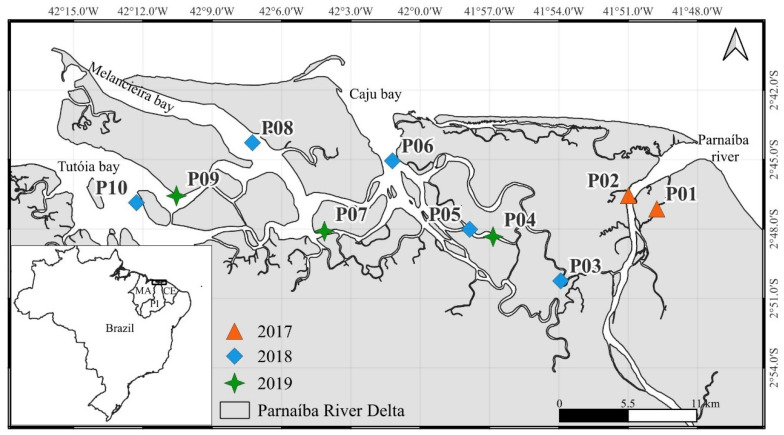
Location of the sampling stations of oysters in the Parnaíba River Delta in Northeast Brazil. The orange triangles indicate the stations sampled in 2017, the blue diamonds the stations sampled in 2018, and the green stars the stations sampled in 2019.

The PRD is the largest open-sea delta of the Americas, located at coordinates 2°39′–2°59′ S and 41°45′–42°12′ W (Figure 1) on the equatorial coast of Brazil (ECB). The PRD includes a 3076 km^2^ very well-preserved mangrove-dominate estuarine area with low population density (~100 hab km^−2^) and human activities, except for tourism and traditional fisheries, with a total production of about 8000 t yr^−1^ [15,28,29]. The main river spans 1485 km and is the second major river of the northeastern Brazilian region, following the São Francisco River. It serves as the border between the states of Piauí and Maranhão until it flows into the Atlantic Ocean. Additionally, the delta area includes the eastern state of Ceará, and the entire PRD is encircled by an Environmental Protection Area (EPA), consisting of a 3138 km^2^ federal conservation unit established by Federal Decree on 28 August 1996, and located at 02°37′–03°05′ S and 42°29′–41°09′ W; it includes the adjacent adjacent continental shelf area [28]. Within the PRD, there are over 1500 km^2^ of pristine mangroves and associated ecosystems. The dominant tree species are *Rhizophora mangle*, *Avicennia germinans*, *A. schaueriana*, and *Laguncularia racemosa*. These mangroves support extensive extractives of crabs and shrimps, clams, and oysters; they also harbor threatened species, such as the red ibis *Eudocimus ruber* [28,30].

The delta has five tidal channels opening into five large bays: Tutóia, Melancieiras, Caju, Canárias, and Igaraçu, with many dozens of islands and tidal creeks. Tidal conditions are mesotidal and semidiurnal, with a 3.3 m range during spring tide [31]. This has a significant influence on the salinity gradient across the delta, varying from 0 to 33 s.p.u. [32,33]. In the PRD artisanal fishery, oyster and crab extraction have significant socioeconomic importance, particularly for low-income populations. However, the anthropogenic activities give rise to socioenvironmental issues, including pollution, unchecked resource exploitation, deforestation, and solid waste disposal from urban areas and from tourism, as well as agriculture, husbandry, aquaculture, and traditional fishing [28,34].

## 2. Materials and Methods

Individuals of *C. rhizophorae* were collected from mangrove roots during low tide in the dry seasons (July to December) of 2017, 2018, and 2019 to minimize the potential seasonal variability of environmental conditions. Samples were taken from 10 stations covering the entire PRD (Figure 1). Due to the complex logistics, longer campaigns covering the entire PRD were unfeasible, as was regular sampling to understand seasonal effects or temporal variations in Hg concentrations related to changing emission loads. Thus, different years resulted in different sample sizes and locations. Samples from all years, including the first pilot sampling in 2018, were included in the present database for analysis. Individuals were collected by hand and placed in ice without depuration, mimicking the harvesting process for human consumption. In the laboratory, individuals were measured and grouped into five shell-height classes (20–40, 41–60, 61–80, 81–100, and 101–120 mm). Composite samples were obtained by pooling 2 to 6 oysters within each size class, totaling 68 samples. Whereas pooling individual oysters of similar size is the practical approach to monitoring and risk assessment studies, the actual individual variability of Hg concentrations, based on pooled samples, has to be discussed with caution. Each sample was weighed before and after lyophilization to estimate water content and to convert Hg concentrations from a dry- to a wet-weight basis. All composite samples are described in Supplementary Material Table S1. Approximately 0.5 g dry oyster soft tissues were digested with 10 mL of concentrated HNO_3_ in Teflon vials in a MARSX-Press microwave digester. Total Hg concentrations were quantified by cold vapor atomic absorption spectrophotometry (CV-AAS) in a Nippon Instrumentation Corp (Kyoto, Japan). (NIC RA-3) spectrophotometer. The digested samples, blanks, and reference material were diluted with Milli-Q water to a final volume of 100 mL. All samples were analyzed in duplicate, with the accepted coefficient of variation (CV) below 15%. The precision and accuracy of the Hg methodology in oysters were tested with certified reference material (Mussel Tissue-ERM CE278K), with a recovery of 101.7 ± 6.3%. The detection limit of the procedure was 0.02 ng g^−1^ and the quantification limit 0.06 ng g^−1^. All Hg concentrations were reported on a wet-weight basis (Table 1), except in Table 2, where wet-weight data were converted to dry-weight data using the measured moisture content in order to compare with data in the literature.

To estimate the exposure risk to Hg through mangrove oyster consumption, we assumed a local ingestion rate (IR_Local_) of 0.013 kg day^−1^, which is the per capita seafood intake estimated for the Piauí State population [35], and an average body weight (BW) of 70 kg for an adult consumer. As this consumption rate includes all types of seafood available for consumers, including oysters, it is likely that the actual oyster-only consumption rate is even lower. Therefore, this is a conservative overestimate of the risk incurred by total seafood consumption. Considering this, and the dietary variability in human populations, we calculated the maximum safe daily ingestion rate (IR_max_) for the average adult consumer and converted these values to number of meals per month (IR_mm_), assuming an average meal size of 150 g. The Hg reference dose (RfD) was 0.0001 mg kg_bw_^−1^ day^−1^ and represents an estimate of daily exposure with no risk of deleterious health effects [36,37]. Exposure risk estimates were calculated according to Bezerra et al. [38] using the following equations:

(1) $${IR}_{max}=\frac{BW\times RfD}{C_{oyster}}$$

where IR_max_ is expressed in kg day ^−1^, and C_oyster_ (mg kg^−1^) is the concentration of Hg in mangrove oysters.

(2) $${IR}_{mm}=\frac{{IR}_{max}\times T_{ap}}{MS}$$

where T_ap_ is the average time period (365.25 days per 12 months or 30.44 days month^−1^), and MS is the meal size (0.150 kg meal^−1^).

We also estimated the daily safe Hg intake (EDI_Hg_ mg kg_BW_^−1^ day^−1^) through oyster consumption and calculated the target hazard quotient (THQ), which represents the health risk from chronic exposure to Hg through oyster consumption, using Equations (3) and (4), respectively.

(3) $${EDI}_{Hg}=\frac{C_{oyster}\times {IR}_{Local}}{BW}$$

(4) $$THQ=\frac{EF\times {IR}_{local}\times ED\times C_{oyster}}{RfD\times BW\times AT}$$

where EF is the exposure frequency (365 days/year), ED is the exposure duration (77 years for the average adult consumer), and AT is the averaging exposure time (EF × ED). A THQ lower than 1 represents no expected health risk, while a THQ higher than 1 represents a potential risk to consumer health.

Normality tests (Kolmogorov–Smirnov) were performed on the biometric data and the Hg concentrations of oysters, both sets of data showing normality (α = 0.05). A Mann–Whitney test was used to compare differences in Hg concentrations between size classes using RStudio version 4.5.1 software [39]. A *p* < 0.05 level of significance was used in all statistical tests.

## 3. Results and Discussion

### 3.1. Total Hg Concentrations in the Mangrove Oysters of the PRD

A total of 68 composite samples of mangrove oysters in the PRD were examined: 57 in 2017, one in 2018, and 10 in 2019. Overall, oyster shell lengths varied from 20 to 117 mm. Most individuals were from classes 20–40 mm (21%), 40–60 mm (36%), and 60–80 mm (27%). The remaining oysters were from classes 80–100 mm (12%) and 100–120 mm (4%) (Supplementary Material Table S1).

**Figure 2:**
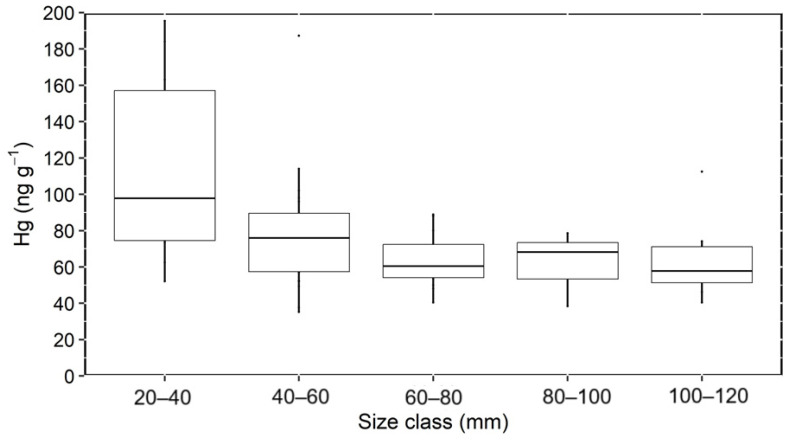
Average Hg concentrations (ng g^−1^ dry weight) in different shell size classes of mangrove oysters from the Parnaíba River Delta.

Overall, Hg concentrations differed significantly among size classes (Kruskal–Wallis rank sum test; α = 0.05) and were higher in the 20–40 mm class compared to the other classes (chi-squared Kruskal–Wallis; *p* < 0.0062). Mangrove oysters between 20 and 40 mm exhibited Hg concentrations varying from 12.5 to 195.4 ng g^−1^, while those in the 40–60 mm class concentrations varied from 9.1 to 114.0 ng g^−1^. In contrast, the three larger size classes (<60 mm) exhibited similarly low Hg concentrations (38.4 to 78.6 ng g^−1^) (Figure 2).

The effect of size on Hg concentrations in oysters is still debatable and varies depending on species and environmental conditions. As it relates to filtering capacity, smaller oysters frequently, but not always, present higher filtering rates than larger individuals [40], which could potentially result in greater Hg uptake. In contrast, smaller oysters may have higher metabolic growth rates, potentially resulting in dilution of incorporated Hg through growth. A high filtering capacity was found in large individuals of *Ostrea edulis*, evidenced by water clearance rates up to five times greater compared to smaller individuals [41]. Although higher filtering rates could lead to greater uptake of metals from the environment, including Hg, the rapid increase in body mass may dilute the concentration of accumulated metals in the tissues of fast-growing oysters. This process explains the relatively lower Hg concentrations in large individuals compared to smaller ones, notwithstanding filtering capacity, still unknown for *C. rhizophorae*.

On the other hand, other factors, such as environmental Hg concentrations and/or bioavailability, can also control Hg incorporation in oysters. Key physical–chemical parameters, such as suspended solids content and salinity, are known to control Hg concentrations in the water column and sediments, which can be associated with oysters’ Hg uptake, as observed in *C. rhizophora* from NE Brazil [25].

The mangrove oyster *C. rhizophorae* prefers filtered particles between 2–10 μm in size and primarily feeds on phytoplankton [19]. In the Parnaíba River, the Chlorophyta division is the dominant phytoplankton group [42]. Selectivity in feeding particles likely contributes to their ability to bioaccumulate the large range of chemical compounds present in their habitats [26], including Hg. Trace metals, including Hg, are commonly associated with small particulate matter in estuarine waters, including phytoplankton and particulate organic matter, as well as silt and clay suspended particles, where Hg may be weakly bound to surface charges and be eventually released when particles are ingested [43,44]. Therefore, Hg adsorbed to large suspended particles may still be taken up, even if oysters do not ingest these particles themselves due to their double-stage filtration systems [40].

Mangrove oysters smaller than 60 mm were collected preferentially in the inner stations of the Parnaíba River, which may be associated with their higher Hg concentrations compared to large ones (>60 mm). On the other hand, larger individuals were observed in stations further seaward and presented lower Hg concentrations. As previously observed, particle filtration is influenced by oyster size; however, local environmental factors, such as salinity, concentration of total suspended solids, and flow rate, may produce spatial and temporal physiological variability in oysters and consequently affect Hg bioaccumulation [40].

Another interesting aspect of Hg accumulation dynamics in oysters is that uptake can be associated with Hg fractionation (i.e., bioavailability) rather than total concentrations [3,45]. In the Jaguaribe River estuary, also on the northeastern coast of Brazil, these authors showed that estuarine biota inhabiting the mixing zone of the estuary, with a greater proportion of Hg in bioavailable fractions (e.g., dissolved and particulate), presented higher Hg concentrations compared to fluvial-influenced zones.

The PRD is under an equatorial climate, with minor season temperature variability (<1 °C), but salinity and suspended solids vary significantly [32,33]. These authors have identified a large salinity gradient (<1–33 p.s.u.) throughout the estuary, including stations where oysters in the present study presented the highest Hg concentrations (Supplementary Material Figure S1). The transition from freshwater to seawater results in a maximum turbidity zone (Supplementary Material Figure S2) with noticeably elevated total suspended particulate levels, which can be as low as 15 mg L^−1^, in the saline end-member, and up to 156 mg L^−1^ in the freshwater end-member. This occurs due to turbulent energy exchanges that resuspend the bottom sediments to the water column and favor the adsorption of elements to fine particles. This process was observed in the PRD by Santos et al. [46], who identified strong adsorption and further deposition of Pb and Cr in the maximum turbidity zone of the PRD. Water and surface sediment throughout the PRD showed a 10-fold variation in total Hg concentration (4.3 to 39.0 pM), mostly as particulate Hg associated with particles (16.03 ± 9.95 pM). Methylmercury (0.04 ± 0.02 pM) represents a minor fraction of the total Hg present in the PRD waters. Seawater intrusion during flood tide is associated with changing Hg concentrations through dilution, whereas particulate Hg accumulates mostly in the fine-grained mangrove sediments (0.14–28.2 ng g^−1^) [12]. Therefore, it is possible that Hg undergoes similar adsorption to suspended particles and becomes more available to the mangrove oysters.

**Figure 3:**
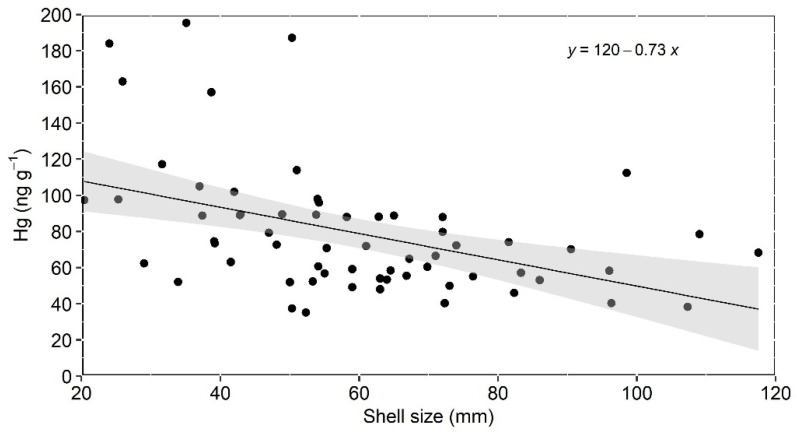
Relation between the concentration of Hg and shell size in mangrove oyster from the Parnaíba River Delta.

Apart from the mentioned stations at the mixing zone, where small oysters dominate, oysters from all other stations exhibited similar and smaller Hg concentrations (Supplementary Material Table S1). This suggests dilution with marine particles and/or Hg desorption from suspended particles with increasing salinity, as suggested by Rodrigues et al. [12]. Desorption was reported to strongly affect metals as the influence of tides and waves increases seaward [46,47]. These processes can decrease the Hg content present in the particulate material filtered by the mangrove oysters. The extremely large variability of Hg concentrations in small oysters (Figure 3), sampled mostly in fluvial-influenced mixing zones, strongly suggests that highly variable salinity and suspended solid contents can influence Hg concentrations. In addition, the faster growth rates of small oysters may result in different Hg contents. Although the relatively small number of samples of each size class hampers a strong statistical analysis, the faster decrease in Hg concentrations with size in the smaller classes of oysters (<60 cm) may corroborate a faster filtration and, therefore, Hg accumulation rates in smaller individuals, but again the small number of samples hampers a more detailed discussion of the topic. However, further studies are necessary to better discern the role played by biological (size) and environmental drivers on Hg oyster concentrations.

Despite the observation of small-sized oysters presenting the highest Hg concentrations and large-sized oysters the lowest, when pooling all size classes, a statistically significant and negative relationship (Pearson, r = −0.4475, *p* < 0.0002) was observed between oyster size and Hg concentration (Figure 3). This correlation, however, disappears when only size classes of >40 mm are considered. Previous studies using only larger *C. rhizophorae* (>40 mm) in other ECB locations also identified no positive significant correlation between Hg concentration and shell size [24,25,48]. The lack of correlation between Hg concentration and shell size may suggest that environmental variables in addition to biological factors influence Hg concentrations in oysters [48]. On the other hand, Vaisman et al. [24] attributed the lack of correlation to the high intrapopulation variability of the Hg concentrations typically observed in many studies. The observed Hg bioaccumulation pattern in the PRD oysters suggests that both biological (size) and the eventual Hg bioavailability in the different sampling sites, e.g., turbidity and salinity variation, also influence Hg content in oysters. It is important to highlight, however, that there is no experimental study, to our knowledge, on the filtration-rate impact of metal uptake by *C. rhizophorae*, so it is not possible to ascertain the relative influence of environmental and biological drivers on Hg bioaccumulation in this oyster species.

**Figure 4:**
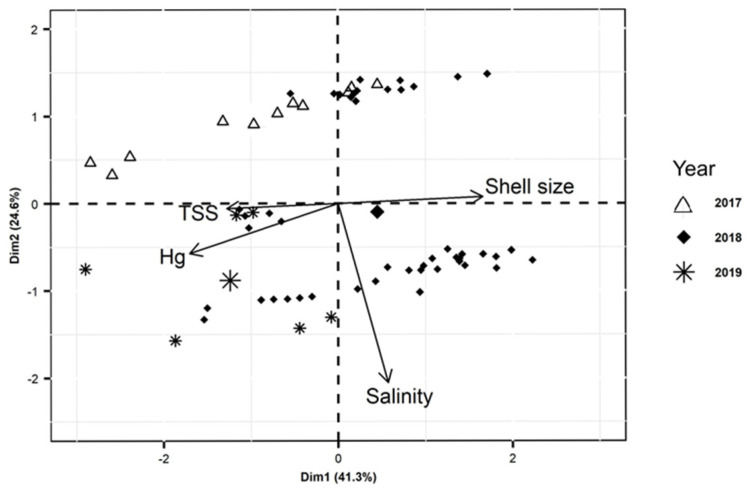
PCA analysis of Hg concentrations, size, and environmental parameters in the Parnaíba River Delta in NE Brazil.

The PCA analysis of Hg concentrations, size, and environmental parameters in the PRD (Figure 4) helps in the interpretation of the relative contribution of the variables upon Hg concentrations in oysters. As expected, Hg concentrations (38.2%), total suspended solid content (TSS) (21.4%), and oyster size (36.1%) show the largest contribution to axis 1 (Dim. 1), whereas salinity (92.6%) contributes to the near totality of the variability of axis 2 (Dim. 2). It is also clear that shell size is diametrically opposed to Hg concentration, confirming the negative correlation between these two variables. The association between salinity and TSS confirms previous observations of low TSS associated with incoming tidal waters [32,33], which also tend to decrease total Hg concentrations in water [12].

The sessile nature of these organisms makes them more susceptible to the variation of element concentrations along the PRD estuarine gradient, which suggests oysters can act as good biological monitors of environmental Hg concentrations. However, to use these oysters as monitoring organisms, the size class needs to be considered.

### 3.2. Concentrations of Hg Across the Biogeographical Distribution of C. rhizophorae

A broad range of Hg concentrations in *C. rhizophorae* is observed along its latitudinal distribution from the Caribbean to South Brazil, and concentrations seem to reflect the general environmental conditions of a given area (Table 1). Sites where point sources are the major source of Hg showed the highest values (up to 1800 ng g^−1^), as on the Sagua la Grande River coast in Cuba [48] and in the Botafogo Estuary and Santa Cruz Canal in Brazil [7,23,49]. These sites received effluents from chlor-alkali plants and are well known for their extremely high Hg environmental levels, resulting in increased Hg concentrations, not only in shellfish, but also in benthic fish (stingrays) [50]. Moderate values are generally observed in industrialized and high-density urban areas along the coast of Brazil. In mangrove areas adjacent to metropolitan areas in Santos Bay and Paranguá Bay in southeastern and South Brazil, respectively, anthropogenic Hg sources include port activities, fertilizer industries, and domestic sewage [22,23]. In the Ceará River estuary in NE Brazil, within the metropolitan area of Fortaleza City, untreated urban effluents from over 1.5 million habitants also contribute to the relatively high Hg concentrations reported in water, sediments, and biota in this river basin [24,25,51]. On the other hand, pristine environments generally show very low Hg concentrations, often lower than 50 ng g^−1^ d.w., as in the Cananeia estuarine complex, Todos os Santos Bay [52], SE Brazil [53] and the Gulf of Paria in the Caribbean [13].

**Table 1:** Mercury (Hg) concentrations reported in mangrove oysters, *Crassostrea rhizophorae*, along their latitudinal distribution from the Caribbean to south Brazil.

Estuary	Shell Size (mm)	Hg Oyster (ng g^−1^) Dry Weight	Article
Parnaíba River Delta-Western ECB	20–40	12.5–195.4	Present study
	40–60	9.1–187.2	
	60–80	5.5–88.8	
	80–100	40.4–112.5	
	100–120	38.4–78.6	
Jaguaribe Estuary-Eastern ECB	<40–60	22–123	[24]
Ceará Estuary-Eastern ECB	<40–60	56–300	
Cocó Estuary-Eastern ECB	<40–60	39–116	
Pacoti Estuary-Eastern ECB	<40–60	21–65	
Ceará Estuary-Eastern ECB	<30/20–40	59.7–96.9	[25]
	>35/40–60	74.9–120.9	
Cocó Estuary-Eastern ECB	<30/20–40	43.9–67.2	
	>35/40–60	53.6–76.3	
Pacoti Estuary-Eastern ECB	<30/20–40	45.1–59.7	
	>35/40–60	40.2–59.4	
Jaguaribe Estuary-Eastern ECB	<30/20–40	55.5–86.0	
	>35/40–60	67.0–85.8	
Ceará Estuary-Eastern ECB	46.3 ± 12.2	38.5–71.0	[26]
Botafogo Estuary-Eastern ECB	40–50	101–1644	[7]
Pernambuco Coast–Eastern ECB	40–80	135–1344	[49]
Piraquê Estuary-Eastern ECB	-	<0.15–87.9	[23]
Santa Cruz Canal Estuarine Complex-Eastern ECB	-	<0.15–1798	
Todos os Santos Bay, Eastern Brazil	-	40–120	[52]
Sepetiba Bay-SE Brazil	31–47	15–23	[19]
Cananeia, estuarine complex, SE Brazil	-	<2–30	[53]
Santos Bay-SE Brazil	-	<0.2–370	[22]
Paranaguá Bay-South Brazil	-	<0.2–350	
Paranaguá Bay-South Brazil	-	63.9–168.7	[23]
Gulf of Paria, Venezuela and Trinidad-Caribbean	40–100	10–70	[13]
Sagua la Grande River and coastal zone, Cuba-Caribbean	-	190–690	[48]

From the Gulf of Paria, between the Venezuela and Trinidad coasts in the Caribbean, as well as throughout the ECB and the NE Brazilian coast, Hg concentrations in the mangrove oysters are lower and less variable, the exception being the Ceará River, in the Metropolitan area of Fortaleza. Throughout the entire ECB extension, it is challenging to associate differences in Hg concentrations with anthropogenic sources. For example, the PRD and the Pacoti Estuary are both protected environmental areas, but their oysters show Hg concentrations as low as those observed in the Jaguaribe River and Cocó River estuaries, which receive inputs from urban areas, agriculture, and aquaculture [24,25]. These variabilities of concentrations probably reflect different Hg bioavailability in response to changing physical–chemical parameters along the estuarine gradient rather than diffuse anthropogenic sources.

However, when comparison is made among estuaries of similar dimensions, but nearly pristine or receiving Hg only from diffuse sources, such as those in on the central Ceará coast (Ceara, Pacoti, Cocó, and Jaguaribe rivers), higher Hg accumulation in oysters is found in urban estuaries (Cocó and Ceará rivers) compared to those in less urbanized estuaries (Pacoti and Jaguaribe rivers) [24,25]. This also seems to be the case for the Pernambuco coast in NE Brazil (135–1344 ng g^−1^) (Table 1), where oysters from pristine, slightly contaminated estuaries, as well as estuaries receiving Hg from industrial point sources, show Hg concentrations varying ten-fold, with the highest ones from estuaries receiving Hg-containing industrial effluents [7,49]. The large PRD extension may eventually include stations with different degrees of Hg input from incipient urbanization and other small Hg sources. This results in high variability Hg content in oysters, with low concentrations in most stations and relatively higher concentrations in those fluvial-influenced stations where Hg is more easily remobilized and has its bioavailability increased, including the formation of methyl-Hg. Although methyl-Hg concentrations are very low in the PRD [12], this Hg species was found to represent 31.9% to 64.5% of the total Hg found in mangrove oysters from southeastern Brazil [19]. Therefore, it is important for future studies to determine the chemical form of Hg in oysters from the PRD, mainly due to the higher concentrations of Hg found in some stations.

In summary, although many studies suggested the use of mangrove oysters, including *C. rhizophorae*, in monitoring programs, the results presented in Table 1 suggest that biomonitoring capacity is limited to the regional scale, where Hg sources are the main driver of accumulation variability. At the local scale (within an estuary, even in large ones such as the PRD), local physicochemistry may be more significant than Hg sources. Therefore, only when the temporal and spatial variability of local physicochemical parameters are well mapped can oysters be used as biological monitors of PRD Hg pollution sources. As the PRD does not have significant anthropogenic Hg sources, the bioaccumulation of Hg in the mangrove oysters probably reflects the higher Hg bioavailability resulting from changes in physicochemical parameters induced by tidal dynamics, particularly in the maximum turbidity zone. Notwithstanding the low anthropogenic pressure in the PRD and the pilot nature of the present study, it is clear that a large variability in Hg concentration occurs. Considering all measured individual values, a range of over five times in Hg concentrations was found, even under the PRD’s pristine conditions; this suggests that Hg can attain relatively high concentrations in oysters in other low-impacted estuaries worldwide. Therefore, further studies in other areas where anthropogenic Hg emissions are low or absent are needed to investigate the spatial and temporal variability of Hg species in abiotic compartments to identify the major factors responsible for localized enrichments and the impact on oysters’ Hg concentrations.

### 3.3. Assessment of Exposure Metrics Through Oyster Consumption According to Brazilian Legislation

The Brazilian National Health Surveillance Agency (ANVISA) establishes the maximum limits for inorganic contaminants in foods, aiming to protect public health. Legislation nº 42, of 29 August 2013 [54] determined a maximum level of 500 ng g^−1^ of total Hg in bivalve mollusks in wet weight.

The Hg concentrations in the PRD oysters were converted to wet weight, considering 80% water content [24], to compare with the Brazilian Legislation (Table 2). The PRD oysters have Hg concentrations one to two orders of magnitude lower than the maximum limits. Considering the potential exposure to Hg in humans consuming oysters from the PRD, we found a very low exposure risk, with an estimated daily Hg ingestion 25 to 50 times lower (Table 2) than the reference dose established by USEPA [35] (RfD = 0.0001 mg kg_bw_^−1^ day^−1^). As a result, the estimated THQ values were lower than 1 for all size classes (Table 2). The calculated maximum safe ingestion rate varied from 0.36 ± 0.14 kg dia^−1^ in the smallest size class (20–40 mm) to 0.60 ± 0.16 kg day^−1^ in the largest size class (80–100 mm). The estimated number of meals per month, on average, was equal to or greater than 74 meals (150 g portion per meal), which shows that the daily or subsistence consumption of oysters from PRD is generally safe as it relates to Hg exposure.

These results can inform fish consumers from other markets in Ceara State and Northeast Brazil. Mercury concentrations in mangrove oysters sampled in the Ceara fish market were also low and posed no risk to local consumers [38]. As most of the mangrove oysters consumed in this market are harvested in the PRD (personal communication), local consumers can be better informed about the generally low risk of exposure to Hg. Another market where oysters are sourced from the PRD is the Sao Luiz fish market. Lacerda et al. [55] estimated that Hg concentrations in fish, associated with a high frequency of consumption, could potentially pose a risk to the sensitive consumer group of children. However, as it relates to mangrove oyster consumption, even a high-frequency consumption would not result in excessive exposure to Hg, as shown in the present study.

It is worth mentioning that, besides Hg contamination, other trace metals were not evaluated in the present study and may eventually raise awareness of high-frequency consumption of oysters from the PRD. For example, Paula Filho et al. [11] and Santos et al. [46] have shown that concentrations of zinc (Zn) in the sediment and suspended solid material could be a matter of concern in the PRD. So, in order to advise on the unrestricted consumption of mangrove oysters from the PRD, it is necessary to assess the concentration of other metals, including Cd, Pb, and Zn, which can bioaccumulate in oysters and other organisms along the food chain at levels potentially harmful to human health.

**Table 2:** Mercury exposure estimates for the consumption of mangrove oysters (ng g^−1^ wet weight) of different shell sizes in the Parnaíba River Delta on the equatorial coast of Brazil. IR_max_—Maximum safe ingestion rate; IR_mm_—Monthly safe number of meals; EDI—Estimated daily Hg ingestion; Target hazard quotient.

Shell Size	Hg Minimum-Maximum (ng g^−1^)	^a^ Hg Mean ± SD	IR Max (kg dia^−1^)	IR Mm (n mes^−1^)	^b^ EDI (mg kg_bw_^−1^ dia^−1^)	THQ
20–40	10.4–39.1	21.2 ± 10.2	0.36 ± 0.14	74 ± 29	<0.0001	<1
40–60	7.0–37.4	14.6 ± 7.1	0.51 ± 0.19	104 ± 19	<0.0001	<1
60–80	8.1–17.76	12.2 ± 3.9	0.57 ± 0.13	116 ± 26	<0.0001	<1
80–100	8.1–22.5	12.8 ± 4.2	0.60 ± 0.16	121 ± 33	<0.0001	<1
100–120	7.7–15.7	12.4 ± 3.4	0.62 ± 0.21	126 ± 42	<0.0001	<1

^a^ Maximum Hg values according to ANVISA nº 42/2013 is 500 ng g^−1^ w.w. [53]. ^b^ EDI compares to the maximum daily reference dose (RfD) for Hg, 0.0001 mg kg_bw_^−1^ day^−1^ [36].

## 4. Conclusions

The present study confirmed that the mangrove oyster, *C. rhizophorae*, is an effective biomonitor of Hg at a regional scale, but at the local (within estuary) scale, local physicochemical dynamics may be more significant than Hg sources, thus requiring a detailed mapping of the spatial and temporal variability of environmental parameters to distinguish between natural and anthropogenic drivers of Hg concentration variations in oysters and thus allows their use as local biomonitors. As the PRD does not have significant anthropogenic Hg sources, the bioaccumulation of Hg in the mangrove oysters reflects the high natural Hg availability in the environment. Further studies are needed to investigate the partitioning of Hg in abiotic compartments to identify possible enrichment in the environment in recent years. Although the Hg concentrations in oysters are acceptable for human consumption, ongoing vigilance and proactive measures are crucial to preserving environmental quality. So, it is also necessary for future research to monitor multiple contaminants to better understand the health risks associated with consuming seafood from the coastal environments on the equatorial coast of Brazil. The present study offers essential information regarding the environmental health of the PRD relative to Hg concentrations in oysters, contributing to the understanding of Hg dynamics in this distinctive and relatively pristine ecosystem. The results could guide conservation efforts and policymaking to maintain the ecological integrity and safety of this important coastal environment.

## Data Availability

The authors declare that the data supporting the findings of this study are available within the paper and its Supplementary Information files. Should any raw data files be needed in another format, they are available from the corresponding author upon reasonable request.

## Supplementary Materials

The following supporting information can be downloaded at: https://www.mdpi.com/article/10.3390/toxics13080678/s1. Table S1: Environmental parameters and biometric information of *Crassostrea rhizophorae* species collected in different years (2017, 2018, and 2019) in the Parnaíba River Delta. TSS means total suspended solids. Figure S1: Salinity distribution in the PRD during the three sampling campaigns. Figure S2: Total suspended solids (TSS) distribution in the PRD during the three sampling campaigns.
