# Supplementary material for: Mercury Bioaccumulation in Mangrove Oysters (Crassostrea rhizophorae) (Guilding, 1828) and Associated Human Exposure from the Parnaíba River Delta, Equatorial Coast of Brazil
Source: Toxics. 2025 Aug 14;13(8):678. doi: 10.3390/toxics13080678 (PMC12390040; doi:10.3390/toxics13080678)

Table S1. Environmental parameters and biometric information of *Crassostrea rhizophorae* species collected in different years (2017, 2018 and 2019) in the Parnaíba River Delta. TSS means total suspended solids.

**Stations**	**Year**	**Musculature**	**Salinity**	**TSS (mg L^{-1})**	**Shell size class (mm)**	**Shell size (mm)**	**Hg (ng g^{-1} w.w.)**
P01	2017	Total	0.740	35.17	20-40	39.2	73.5
P01	2017	Total			20-40	25.3	97.8
P01	2017	Total			40-60	59.0	49.3
P01	2017	Total			40-60	50.0	52.0
P01	2017	Total			40-60	54.1	60.7
P01	2017	Total			40-60	47.0	79.3
P01	2017	Total			40-60	42.9	89.6
P02	2017	Total	33.85	20-40	20-40	38.7	157.1
P02	2017	Total			20-40	25.9	163.0
P02	2017	Total			20-40	35.1	195.4
P02	2017	Total			40-60	50.3	187.2
P03	2018	Total	1.1	47.56	20-40	33.9	52.1
P03	2018	Total			40-60	52.3	35.2
P03	2018	Total			40-60	55.0	56.8
P03	2018	Total			40-60	59.0	59.2
P03	2018	Total			60-80	72.3	40.4
P03	2018	Total			60-80	63.0	54.1
P03	2018	Total			60-80	76.4	55.2
P03	2018	Total			60-80	64.5	58.5
P03	2018	Total			60-80	67.2	64.9
P03	2018	Total			60-80	74.0	72.4
P03	2018	Total			80-100	96.3	40.4
P03	2018	Total			80-100	86.0	53.1
P03	2018	Total			80-100	83.3	57.2
P03	2018	Total			100-120	107.3	38.4
P04	2019	Total	15.3	47.67	20-40	37	105.0
P04	2019	Total			20-40	24	184.0
P04	2019	Total			40-60	42	102.0
P05	2018	Total			20-40	29.0	62.4
P05	2018	Total			20-40	39.1	74.6
P05	2018	Total			40-60	48.1	72.8
P05	2018	Total			40-60	54.2	96.0

P05	2018	Total	18.3	73.00	60-80	62.8	88.2
P06	2018	Total	30.7	59.27	20-40	37.4	88.8
P06	2018	Total			20-40	20.4	97.4
P06	2018	Total			20-40	31.6	117.2
P06	2018	Total			40-60	58.2	88.1
P06	2018	Total			40-60	42.8	89.1
P06	2018	Total			40-60	53.8	89.3
P06	2018	Total			40-60	48.9	89.6
P06	2018	Total			60-80	72.0	79.9
P07	2019	Total			40-60	54	98.0
P07	2019	Total			40-60	51	114.0
P08	2018	Total	29	15.60	40-60	50.3	37.5
P08	2018	Total			40-60	53.3	52.4
P08	2018	Total			40-60	41.5	63.2
P08	2018	Total			40-60	55.3	70.9
P08	2018	Total			60-80	63.0	48.1
P08	2018	Total			60-80	73.0	50.0
P08	2018	Total			60-80	64.0	53.4
P08	2018	Total			60-80	66.8	55.6
P08	2018	Total			60-80	69.8	60.5
P08	2018	Total			60-80	61.0	72.0
P08	2018	Total			80-100	82.3	46.1
P09	2019	Total	36.5	156.25	60-80	72	88.0
P10	2018	Total	28.8	31.56	60-80	71.0	66.6
P10	2018	Total			60-80	65.0	88.8
P10	2018	Total			80-100	96.0	58.4
P10	2018	Total			80-100	90.5	70.2
P10	2018	Total			80-100	81.5	74.1
P10	2018	Total			80-100	98.5	112.5
P10	2018	Total			100-120	117.5	68.3
P10	2018	Total			100-120	109.0	78.6

Figure S1. Salinity distribution in the PRD during the three sampling campaigns.

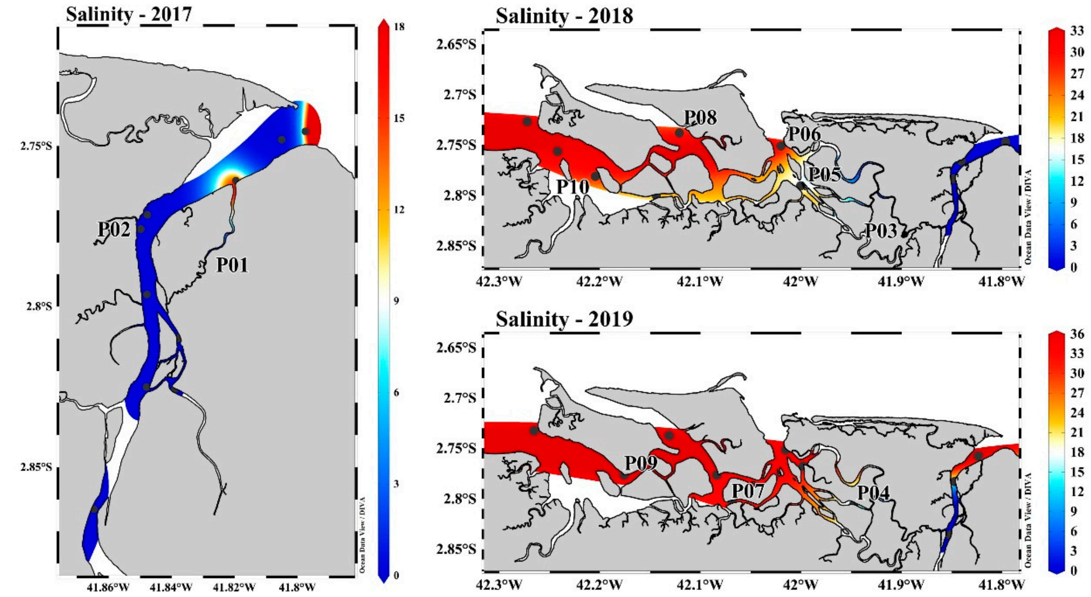

Figure S2. Total suspended solids (TSS) distribution in the PRD during the three sampling campaigns.

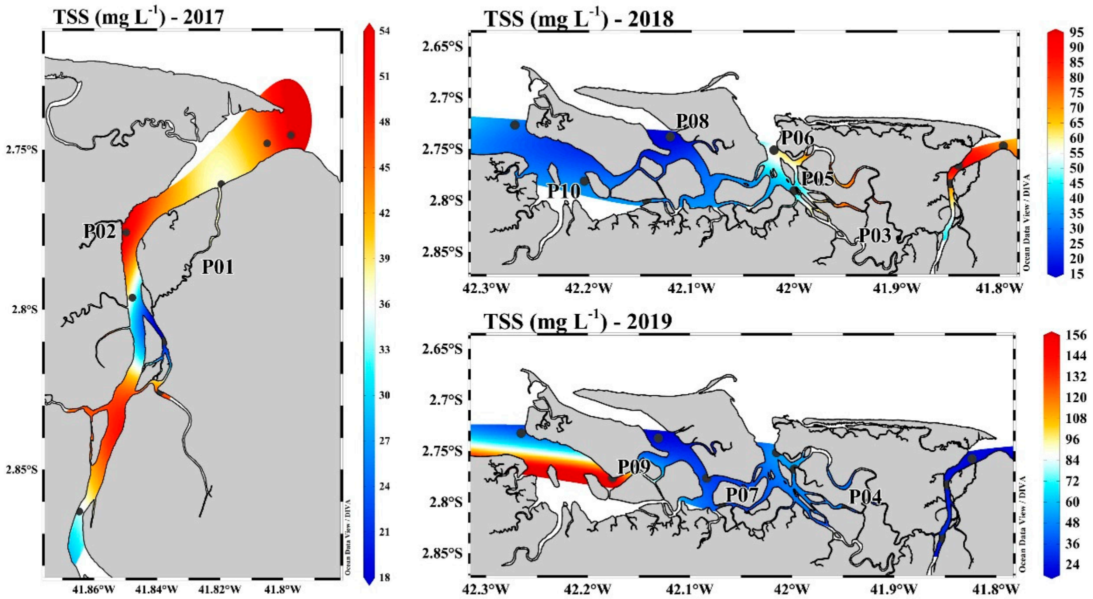

Supplement: Supplementary file 1 [file toxics-13-00678-s001.zip › toxics-3762812-supplementary.pdf]
